# Case report: Rare lung disease of infancy diagnosed with the assistance of a home pulse oximetry baby monitor

**DOI:** 10.3389/fped.2022.918764

**Published:** 2022-09-06

**Authors:** Kevin H. Yang, Art Kulatti, Kimberly Sherer, Aparna Rao, Mateja Cernelc-Kohan

**Affiliations:** ^1^Rady Children’s Hospital, UC San Diego School of Medicine, San Diego, CA, United States; ^2^Division of Respiratory Medicine, Rady Children’s Hospital, San Diego, CA, United States

**Keywords:** NEHI, pulmonology, bronchiolitis, asthma, medical technology, case report, pulse oximetry

## Abstract

Neuroendocrine cell hyperplasia of infancy (NEHI) is a rare childhood interstitial lung disease characterized by a gradual onset of tachypnea, hypoxemia, and failure to thrive in the first 2 years of life. NEHI is challenging to diagnose and can masquerade as common respiratory infections and reactive airway disease. Timely diagnosis is essential to optimize management of comorbidities, improve outcomes, and prevent unnecessary interventions. We report a case of a 14-month-old male who was hospitalized multiple times with recurrent episodes of presumed bronchiolitis. However, early on, the parents had detected unexplained nighttime hypoxemia with a wearable home pulse oximetry baby monitor. While recurrent respiratory infections are common in infancy, our patient had numerous persistent symptoms refractory to traditional treatments, which prompted further workup and ultimately led to the diagnosis of NEHI. The home baby monitor provided useful information that accelerated workup for a presentation that did not fit the usual picture of recurrent bronchiolitis, bronchospasm, or pneumonia. These devices that monitor infant cardiopulmonary status and oxygenation are becoming increasingly popular for home use. There is controversy over their clinical utility due to the frequency of false alarms, excessive parental reliance on these devices, and lack of Food and Drug Administration oversight to ensure accuracy and effectiveness of these devices. Our case provides an example of how in certain clinical settings, information from these devices might serve as a complementary tool in the pediatrician’s medical decision-making and possibly lead to a rare diagnosis such as NEHI.

## Introduction

Childhood interstitial lung diseases (chILD) are a group of rare diseases that can be difficult to diagnose and often pose a clinical challenge to the general pediatrician. Among the chILD disorders, neuroendocrine cell hyperplasia of infancy (NEHI) is a diffuse lung disease first described in 2005 and previously known as persistent tachypnea of infancy (1). Since then, numerous case reports have documented the symptoms associated with NEHI, characterized by a gradual onset of tachypnea, hypoxemia, and failure to thrive seen in the first 2 years of life (1–8).

The etiology of NEHI is unknown, although genetic causes are thought to play a role (9, 10). Males seem to be affected more than females (1, 11). One hypothesis suggests that overactive pulmonary neuroendocrine cells (PNECs) produce bioactive peptides including serotonin, gastrin releasing peptide, cholecystokinin, and calcitonin gene-related peptide that result in bronchiolar constriction (12). Infant pulmonary function testing data has suggested that significant airflow limitation and air trapping may be partially responsible for pulmonary exacerbations (13–15).

Lung biopsy demonstrating increased numbers of PNECs within bronchioles and alveolar ducts without evidence of other abnormalities has historically been the diagnostic gold standard for NEHI (16, 17). However, computerized tomography (CT) of the chest has been increasingly used as a non-invasive test to support clinical suspicion given its relatively high sensitivity and specificity (78 and 100%, respectively) (18, 19). Current guidelines from the American Thoracic Society recommend the use of CT scanning without lung biopsy for diagnosis of chILD except in patients with atypical clinical or CT findings (20). Characteristic CT findings include ground-glass opacification confined to the middle lobe, lingula, and paramediastinal regions of the lungs, in addition to mosaic areas of air-trapping (4, 19).

The accurate characterization of NEHI is critical given its implications for patient management and prognosis. The treatment for NEHI is primarily supportive, involving oxygen therapy and adjunctive therapies for associated comorbidities, which include gastroesophageal reflux, aspiration, poor growth, developmental delay, immune system abnormalities, and sleep apnea (2, 3, 8, 11, 21, 22). Previous case reports have documented repeated hospitalizations, numerous comorbidities, and unnecessary treatments due to delayed diagnoses of NEHI given its similarity in presentation to other respiratory illnesses in infancy (2–4, 6, 7). For these reasons, increased awareness among pediatricians of NEHI’s clinical presentation and its mimickers may provide timely diagnosis and improvements in disease management and prognosis. In addition, improvements in technology to better detect signs of hypoxemia or tachypnea in NEHI may help expedite diagnostic workup.

**FIGURE 1:**
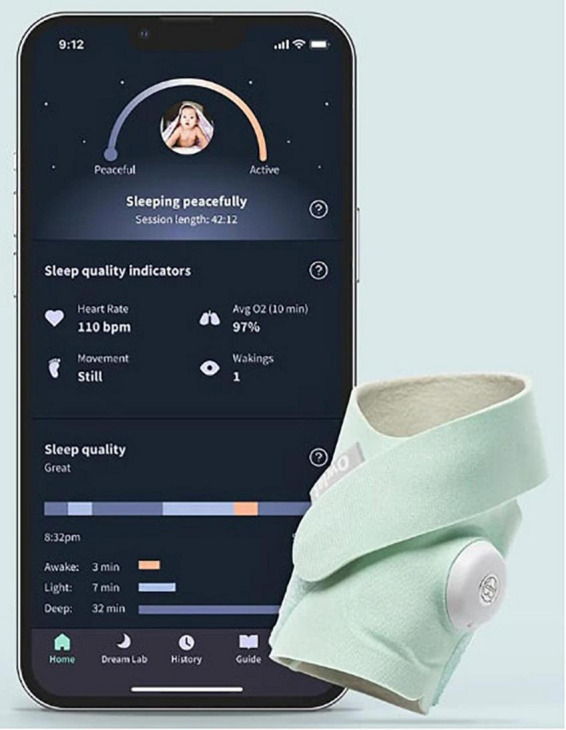
Example of a wearable home pulse oximetry baby monitor and associated mobile application. With copyright permission from Owlet Baby Care.

One such example of technology that has this potential is the increasingly popular commercial “smart” home baby monitors (23, 24). Sensors on these devices are integrated with smartphone applications to record and alert parents of any abnormalities in their infant’s respirations, pulse, and blood oxygen saturation (Figure 1). We report a case in which nighttime hypoxemia detected by a “smart” home baby monitor prompted the pediatrician to pursue further diagnostic workup which ultimately resulted in the diagnosis of NEHI.

## Case presentation

A 14-month-old male of Asian and Caucasian descent presented to our institution with an 8-month history of recurrent cough, tachypnea, and hypoxemia, requiring repeated hospital admissions. He was a full-term infant with an uncomplicated neonatal course and was born average size for gestational age. His early infancy was unremarkable aside from mild eczema, and he received all routine vaccinations. His family history was negative for significant medical or genetic conditions. He lived with both parents at home and was not taking any medications. A brief timeline summary of relevant events and interventions are presented in Table 1.

**TABLE 1:** Timeline of relevant events and interventions leading up to diagnosis of NEHI.

Date	Event	Diagnostics	Treatment/intervention
06/09/2020	Office visit for 2 weeks of dry cough and nighttime hypoxemia (SpO_2_ 83–96% on home baby monitor).	Physical exam (PEX) notable for tachypnea, subcostal retractions, decreased breath sounds, mild scattered wheezes, and bilateral crackles. Diagnosed with bronchiolitis and superimposed pneumonia.	Amoxicillin (ineffective) Albuterol (ineffective)
06/19/2020	Inpatient hospitalization overnight for continued respiratory symptoms and SpO_2_ in mid 80s% at night on baby monitor.	PEX notable for subcostal and intercostal retractions and tachypneic to 80s. O_2_ saturations in low SpO_2_ 90%. CXR with patchy opacity in right mid-lung field. Admitted overnight for respiratory distress and suspected reactive airway disease.	Dexamethasone (moderately effective) Albuterol (ineffective)
6/25/2020	Inpatient hospitalization x2 nights for continued symptoms despite use of bronchodilators and antibiotics.	PEX notable for tachypnea to 66 with suprasternal retractions, O_2_ saturations in mid to low SpO_2_ 90%, and rales/crackles of RLL. CXR normal. Respiratory viral panel negative. Labs normal. Echocardiogram normal. Working diagnosis: recurrent bronchiolitis.	Dexamethasone (effective) Albuterol (ineffective) Pulmicort (ineffective) Referral to allergy and immunology
11/2020	Emergency department for shortness of breath and nighttime hypoxemia to SpO_2_ 85% on home baby monitor.	CXR with bilateral patchy perihilar opacities. Diagnosed with pneumonia.	Dexamethasone (effective) Antibiotics (effective)
01/2021	Sleep medicine specialist visit for concern for sleep-disordered breathing.	Polysomnography with evidence of OSA with OAHI of 5.5. SpO_2_ nadir: 87%, SpO_2_ desaturations < 94% were observed 20% of total sleep time.	Supplemental O_2_ 0.5 L at night (effective) Referral to ENT specialist
02/2021 and 03/2021	Office visits with pediatric pulmonologist and hospitalization for diagnostic workup.	CXR with patchy ill-defined perihilar opacities. Sweat chloride test normal. Immune work-up unremarkable. Bronchoscopy with bronchoalveolar lavage normal. High-resolution CT scan consistent with diagnosis of NEHI.	Supplemental O_2_ 1 L at night (effective) Referral to speech therapy and nutrition

**FIGURE 2:**
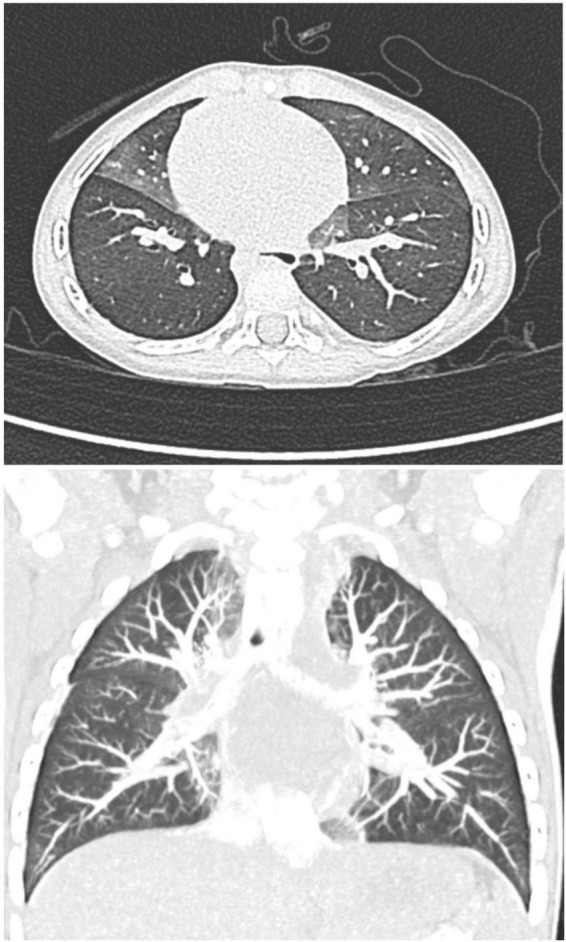
Axial and coronal high-resolution CT chest, demonstrating characteristic geographic ground-glass opacities centrally and most prominently in the right middle lobe and lingula, highly consistent with NEHI.

He first presented to the pediatrician’s office at 6 months of age for 2 weeks of dry cough and hypoxemia. The parents had recently bought a consumer-grade pulse oximetry-based home baby monitoring device and noticed low oxygen saturations (SpO_2_: 83–91%) on the device at night. Exam was notable for tachypnea, subcostal retractions, decreased breath sounds, mild scattered wheezes, and bilateral crackles. No fevers were reported. He was diagnosed with bronchiolitis and superimposed pneumonia and was prescribed antibiotics. Within a few weeks of initial presentation, he was hospitalized twice for persistent symptoms of tachypnea and hypoxemia (both during the day and night) with presumed diagnoses of recurrent bronchiolitis and reactive airway disease. He was treated with supplemental oxygen, albuterol, and oral and inhaled steroids. Chest x-ray (CXR) showed a patchy opacity in the right mid-lung field. Respiratory viral panels were negative. Echocardiogram was normal. He continued to have persistent tachypnea, retractions, and crackles on exam outside of acute respiratory illnesses, and his parents continued to report hypoxemia at night on their home monitor. These concerns along with his poor weight gain prompted formal polysomnography. The sleep study showed obstructive sleep apnea (Apnea Hypopnea Index of 5.5) and episodes of persistent hypoxemia below SpO2 90%, and he was referred to a pediatric pulmonologist. Repeat CXR showed patchy ill-defined perihilar opacities. Sweat chloride test was normal and immune work-up was unrevealing. A bronchoscopy with bronchoalveolar lavage was normal. He ultimately underwent a high-resolution CT scan which showed areas of mosaic attenuation and geographic ground-glass opacities in a predominant perihilar distribution, supporting the diagnosis of NEHI (Figure 2).

**FIGURE 3:**
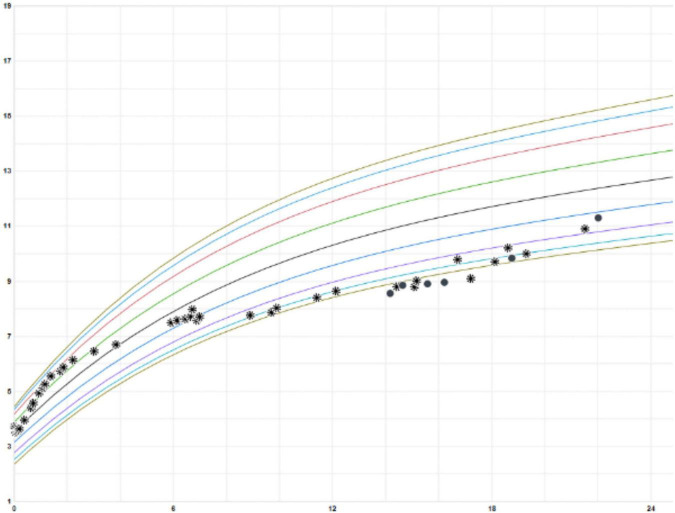
Growth chart of patient’s weight (kilograms) over age (months).

He was started on 1 L supplemental oxygen at night which resolved his nocturnal hypoxemia. He was followed by pulmonology, speech therapy (for language delay), and nutrition consistently. He exhibited gradual improvement at follow-up visits. His inhaled bronchodilators and steroids were weaned off by age 18 months. Currently, at age 2 years, his tachypnea and retractions have improved. He has had good catch-up growth (Figure 3), is making developmental strides, and has not had any additional pulmonary exacerbations or hospitalizations.

## Discussion

Our patient presented with the classic non-specific symptoms of NEHI described in prior case reports (1–8) including tachypnea, hypoxemia, retractions, and crackles on exam with poor weight gain related to increased caloric demands. Working diagnoses included prolonged and recurrent viral bronchiolitis as well as pneumonia, reactive airway disease, and obstructive sleep apnea. Admittedly, there was reported wheezing, which is not typical for NEHI but may occur. However, his symptoms were inconsistently responsive to bronchodilators, glucocorticoids, and antibiotics, as seen in prior case reports (1, 2, 5, 25), thereby making the typical common respiratory diseases less likely.

Fortunately, prospective studies on children with NEHI demonstrate gradual improvement in symptoms over time, with associated decreased oxygen requirements (1, 8, 13, 25, 26). This was observed in our patient over subsequent months, with resultant weaning of bronchodilators and steroids and only 0.5 L of oxygen required at nighttime. However, viral respiratory illnesses can be more severe in NEHI patients, causing NEHI “exacerbations” as seen in our case. Additionally, the aforementioned comorbidities often require subspecialty management and therefore require prompt screening and evaluation. One common clinical finding associated with NEHI that warrants further research is hypoxemia. While hypoxemia is common, the amount of oxygen needed commonly varies among children with NEHI. Some require oxygen 24 h a day, while others only require supplemental oxygen at night and during NEHI exacerbations. Most NEHI patients eventually decrease and grow out of the need for supplementation, although some may require oxygen supplementation for years. Reasons for these differences remain unclear and warrant further investigation with regard to the relationship between hypoxemia and NEHI as well as other factors that may be associated with hypoxemia in this group such as circadian rhythm dysregulation and vagal nerve stimulation (27, 28).

The persistent nocturnal hypoxemia detected by the home baby monitor in this case provided useful data that prompted and accelerated further workup for a presentation that did not fit the usual characteristics of recurrent bronchiolitis. These devices have increased significantly in popularity in the past few years, and analysts have predicted that the “smart” home baby monitor market is expected to grow to $1.8 billion by 2028 (29). Parents were surveyed regarding reasons for purchasing these devices, with 75% endorsing their use for “peace of mind,” and 94% reporting better quality of sleep as a result of using these devices (30).

Despite the increasing popularity of these monitors, there exists controversary over the clinical utility of these devices, especially in healthy, term infants (31). Critics express concerns that there will inevitably be false alarms that may lead to unnecessary hospitalizations or overdiagnoses (23). An American Academy of Pediatrics policy statement in 2016 recommended against the use of these monitors as a strategy to reduce risk of Sudden Infant Death Syndrome (SIDS) (32). Furthermore, one of the major obstacles against more regular use of these devices is the current lack of regulation by the Food and Drug Administration (FDA) to ensure safety, accuracy, and effectiveness of these devices. On the other hand, proponents suggest that these devices can provide parents and clinicians with valuable information about the infant. Some clinicians argue that in select populations, including premature infants at higher risk of recurrent apnea, bradycardia, and hypoxemia, physiologic home monitoring may be indicated (23).

Although there is controversy over the use of home baby monitors, the literature surrounding the clinical utility of these monitors is continuing to evolve. A 2018 study compared two commercial home baby monitors to an FDA-approved pulse oximeter and found that one of them did not detect hypoxemia while the other performed inconsistently. These authors reported the sensitivity and specificity for the latter to detect hypoxemia as 88.8 and 85.7%, respectively (24). Another study found that clinically significant alerts for some of these devices tended to be clustered and repeated, thereby increasing credibility of alerts (30). Additionally, Browne et al. (33) tested an Android smartphone with an embedded photoplethysmography biosensor and application that measures heart rate and blood oxygen saturation. The authors found these smartphone sensors to perform with similar accuracy, precision, and reliability to hospital reference devices, and suggested that these applications should undergo FDA approval to be used for clinical purposes. In the era of the COVID-19 pandemic and with the expansion of telemedicine, the accessibility and advancement in home monitoring devices may be of value for monitoring infants with chronic lung disease.

The observations in our case report should be interpreted in the context of several limitations. First, this report was limited by the small sample size and may not be generalizable or representative of all cases of NEHI. Second, some aspects of the case presentation were based on parental self-report. For example, the oxygen saturation readings from the baby monitor were self-reported as we did not extract the data from the mobile application for verification. However, the patient’s sleep study confirmed evidence of nocturnal hypoxemia below SpO_2_ 90% along with low saturation readings during his hospitalization. Third, we did not perform a lung biopsy for confirmation of diagnosis of NEHI, as current guidelines recommend against the use of lung biopsy except in patients with atypical clinical or CT findings (20). We also did not assess for increased peptide levels (i.e., gastric releasing peptide). Elevations of these peptides can be expected in NEHI from overactive PNECs; however, the application of these levels is a more recently developed concept that is still actively undergoing research (20, 34). Future studies may benefit from measuring peptide levels in these patients to further elucidate the pathophysiology of NEHI.

In light of the aforementioned limitations, this report highlights a case in which use of a home baby monitor helped with diagnosis of a rare lung disease. Because use of these monitors has grown significantly, one of the biggest challenges posed by these “smart” home baby monitors is interpretation of the large amount of data they generate (30). For parents, abnormal values may generate worry, while for providers, it may be challenging to address every abnormal result. However, as these monitors continue to improve in accuracy and grow in popularity, pediatricians will be presented with biometric data that have the potential to hasten diagnosis of rare diseases, including NEHI. Our case demonstrates how information from these devices might serve as complementary tools for pediatricians in settings where a patient’s presentation may be complex or rare. Future studies are therefore warranted to better understand the accuracy and utility of these “smart” home baby monitors.

## Data availability statement

The original contributions presented in the study are included in the article/supplementary material, further inquiries can be directed to the corresponding author.

## Ethics statement

Ethical review and approval was not required for the study on human participants in accordance with the local legislation and institutional requirements. Written informed consent to participate in this study was provided by the participants’ legal guardian/next of kin. Written informed consent was obtained from the minor(s)’ legal guardian/next of kin for the publication of any potentially identifiable images or data included in this article.

## Author contributions

KY performed the literature search, reviewed the medical records, drafted the initial manuscript, and reviewed and revised the manuscript. AK and KS conceptualized and designed the study, coordinated and supervised the data collection, and reviewed and revised the initial and final manuscript. MC-K and AR conceptualized and designed the study and critically reviewed and edited the manuscript for important intellectual content. All authors approved the final manuscript as submitted and agreed to be accountable for all aspects of the work.
